# Perceptions of X+Y Scheduling Among Combined Internal Medicine-Pediatrics Residency Trainees: A Qualitative Program Evaluation

**DOI:** 10.7759/cureus.52983

**Published:** 2024-01-26

**Authors:** Dava Szalda, Nathan R Stehouwer, Jennifer B Walsh, Kathryn Diamond-Falk, Bhavesh Patel, Hillary Spangler, Mridula Nadamuni, Michael Contarino

**Affiliations:** 1 Internal Medicine-Pediatrics, Children's Hospital of Philadelphia, Philadelphia, USA; 2 Internal Medicine-Pediatrics, Case Western Reserve University School of Medicine, Cleveland, USA; 3 Internal Medicine-Pediatrics, University of Texas Southwestern Medical Center, Dallas, USA; 4 Internal Medicine-Pediatrics, Maine Medical Center, Portland, USA; 5 Internal Medicine-Pediatrics, University of North Carolina at Chapel Hill, Chapel Hill, USA

**Keywords:** med-peds residency, resident continuity clinic, med-peds resident education, med-peds, x + y scheduling

## Abstract

Objective: The study aims to elicit perceived benefits and downsides of X+Y scheduling for combined Internal Medicine-Pediatrics (Med-Peds) residents via focus groups.

Methods: Five focus groups were conducted with Med-Peds residents in participating programs which utilized X+Y scheduling. Onefocus group was held per participating institution. Each focus group was facilitated by a chief resident from a different participating institution. Questions were developed by the study team after a review of the literature and local experience with X+Y scheduling and included open-ended questions. Focus groups were recorded and transcribed. Transcripts were reviewed by study team members, and representative themes and quotes were presented. The main outcome was to evaluate the perceived benefits and downsides of X+Y scheduling for Med-Peds.

Results: Results from four of the five focus groups were fully reviewed. Themes regarding the benefits of X+Y scheduling included (1) improved inpatient and outpatient experience, (2) predictability in schedule which improved wellness, and (3) longitudinal time for career exploration. Downsides of X+Y scheduling were highlighted as well including (1) condensing too many experiences into Y time and (2) challenges that exist when categorical medicine and pediatrics programs use different block schedules.

Conclusions: X+Y schedules create potential solutions for longstanding barriers to medical education and notably conflict with inpatient and outpatient responsibilities. Our data shows similar benefits to X+Y scheduling for combined residents as for their categorical colleagues and sheds light on some unique considerations for combined programs and trainees. Additional studies should continue to assess the effect of X+Y scheduling on our combined trainees.

## Introduction

X+Y scheduling (where X refers to rotations without continuity clinic and Y refers to ambulatory blocks with continuity clinic) has become a valuable option for residency scheduling with the Accreditation Council for Graduate Medical Education (ACGME) requirements that require minimizing conflicting inpatient and outpatient responsibilities and increasing ambulatory training time [1]. X+Y scheduling was first adopted in Internal Medicine residency programs [2,3]. Participating program leaders have described an improved ability to focus on current rotations, improved satisfaction with continuity clinic, and improved ability to deliver a consistent curriculum with X+Y scheduling [4]. Internal Medicine residents report improved satisfaction and an improvement in the conflict between inpatient and outpatient responsibilities [5]. Reports on the impact of X+Y scheduling on ambulatory education have shown increased time in the ambulatory setting, improved resident satisfaction with ambulatory time [6], and no significant impact on patient continuity and satisfaction [5].

More recently, Pediatrics residency programs adopted X+Y scheduling through the ACGME’s Advancing Innovation in Residency Education (AIRE) pilot, waiving the previous requirement that mandated continuity clinic experiences be scheduled over a minimum of 26 weeks per year (Program Requirement IV.A.6.b, ACGME/AIRE). Initial reports of X+Y scheduling in the Pediatrics residency program suggest an overall positive resident experience. Specifically, evaluations in Pediatrics programs revealed perceived improvements in outpatient continuity, inpatient workflow, and overall job stress and mixed effects on continuity and follow-up in the outpatient setting [7,8].

With the increased implementation of X+Y scheduling in Pediatrics and Internal Medicine programs, combined Internal Medicine-Pediatrics (Med-Peds) programs have a greater opportunity to fully participate in X+Y scheduling; however, no published data exists on Med-Peds residents’ perceptions of X+Y scheduling. Limited Med-Peds-specific data from the pediatric AIRE study (personal communication, Joanna Lewis) suggested that the Med-Peds residents who were identified in the sample (n=8) responded similarly to categorical colleagues in terms of preference for X+Y scheduling over traditional scheduling (85.7%), allowing for teaching outside of inpatient rounds (62.5%), and satisfaction with continuity clinic scheduling (75%). However, there are often specific considerations when adapting schedules to combined Med-Peds training programs, including ACGME requirements and clinic considerations. New schedules often have unintended consequences and vary by individual program circumstances. The specific aim of this project was to evaluate the perceived effects of X+Y scheduling for combined Med-Peds residents via qualitative data collected by focus groups. Qualitative assessment is a useful tool to understand aspects of X+Y scheduling that were particularly effective or problematic for combined trainees and to inform future quantitative assessments on themes uncovered. Qualitative assessment was selected here as a way to better assess the aspects of X+Y scheduling that impact Med-Peds residents while incorporating the diversity of individual program circumstances.

## Materials and methods

The Pediatric AIRE pilot facilitated the implementation of X+Y scheduling across multiple Pediatrics and subsequently five Med-Peds residency programs. Each Med-Peds program developed their own nuanced X+Y schedule independent from each other but in collaboration with their categorical programs, and therefore, they have varying X+Y structures, including 4+1 and 6+2, and varied experiences in the outpatient curriculum (for example, combined vs. separate Med-Peds clinics). Importantly, X+Y scheduling for all programs, whether Internal Medicine, Pediatrics, or Med-Peds, eliminated the need for residents to be scheduled for continuity clinics while on inpatient rotations, removing the resulting handoffs of patient care on clinic days.

In April and May of 2022, Med-Peds residents from the five Med-Peds residency programs participating in X+Y scheduling were invited to participate in focus groups regarding their perceptions of X+Y scheduling. Five 60-minute focus groups were conducted. Each participating institution had one focus group with a convenience sample of residents who were attending the Med-Peds conference due to the small available sample size (about 16 residents per program, five to 10 residents in each conference/focus group). In terms of representation, each focus group included residents of varying years and experience with X+Y scheduling. Focus groups were facilitated by a chief resident from a different participating institution, rather than their own institution, to reduce bias and leading follow-up questions. Questions were developed by the study team after a review of the literature and local experience with X+Y scheduling [4-8]. The focus group guide included open-ended questions to evaluate the perceived benefits and downsides of X+Y scheduling specifically for Med-Peds residents, including effects on inpatient and outpatient care, well-being, and mentorship, for example: “What do you think may be unique in the experience of X+Y scheduling for Med-Peds residents in comparison to your categorical colleagues?” The full interview guide is included in this article (see Appendices).

**Figure 1:**
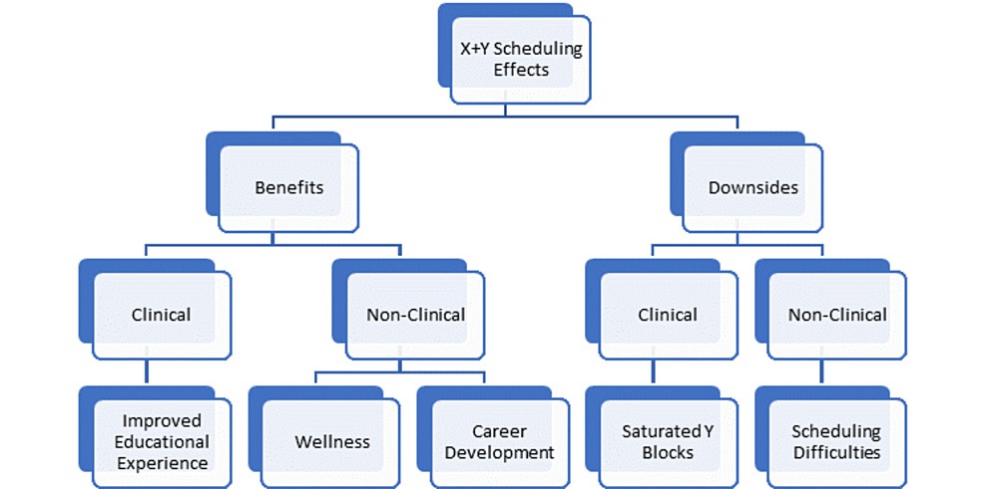
Framework

Each focus group was recorded and transcribed. We used general inductive analysis to identify themes from the focus groups [9]. General inductive analysis was chosen for data analysis as this project centered around program improvement and thus our analysis was guided by evaluation objectives which helped identify domains and topics to be investigated. Three research team members (DS, BP, MN) conducted multiple readings and interpretations of the raw data. Raw data was used to develop a framework of the positive and negative effects of X+Y scheduling, considering direct clinical and non-clinical effects (see Figure 1). After discussion, seven initial effects were collapsed into five, three benefits and two downsides of X+Y scheduling for Med-Peds residencies. These effects will be referred to as themes. Though this project was primarily conducted for program review and improvement, data checking was carried out via member checking and analyst triangulation. Software was not used. Saturation was thought to be reached as no new themes were identified from the final transcript review. Representative verbatim comments were selected for presentation.

This focus group evaluation was reviewed and classified as exempt by the Institutional Review Board at all participating institutions. All participants provided verbal consent at the onset of their focus group.

## Results

Results from four of the five focus groups were reviewed. The transcript from one group was not available for analysis due to a recording malfunction. This focus group was not repeated due to the consistency of results from the other groups and sensitivity to trainees' time.

The following positive themes associated with a change to X+Y scheduling were identified: (1) improved educational experience, (2) enhanced wellness, and (3) increased longitudinal time for career exploration. Challenges included (1) scheduling difficulties due to mismatch between categorical X+Y schedules and (2) oversaturation of the Y block with too many additional experiences. 

Several participants highlighted improved experiences in both the inpatient and outpatient settings. X+Y scheduling led to “decreasing pages when not on the floor” and “decreasing handoffs.” The prior schedule was noted to be “disorienting” at times, shifting abruptly from inpatient work to clinic. Clinic experience was also highlighted as more successful with “consistent preceptors and cohorts.” Clinic didactics were more successful with X+Y scheduling because with traditional scheduling models residents were “rushing there” (to the clinic) and “patients were already there” (waiting to be seen), while X+Y scheduling eliminated transit times from morning inpatient wards to afternoon outpatient clinic. Residents who had experienced X+Y in only one categorical program for some portion of their training expressed the strongest preference for X+Y scheduling.

In terms of wellness, participants cited predictability in schedules as a major advantage to X+Y scheduling. Residents also noted the beneficial elimination of exceedingly long stretches of inpatient rotations. Residents appreciated knowing their less rigorous weeks and free weekends well in advance. In addition, improved emotional exhaustion came in the form of decreased cognitive overload related to not functioning in both inpatient and outpatient roles during the same day. Residents described that while building new knowledge bases and foundations, it is helpful to compartmentalize inpatient and outpatient learning and systems, specifically for learners new to these roles. Residents also cited outpatient activities with built-in half days as an opportunity for time to catch up on personal appointments.

In terms of career development, residents found the predictability and relative flexibility afforded by the Y block to improve career development via additional clinic experiences, mentorship, and participation in extracurricular projects. The potential for elective half days during the Y block time was seen as a benefit for Med-Peds residents who may have more limited elective time in the setting of more required rotations compared with categorical (Internal Medicine and Pediatric) resident training requirements based on ACGME guidelines. Residents cite a better ability to plan mentor meetings with predictability in their schedule. Extracurricular projects were also perceived to be more successful with time in longitudinal Y blocks that are “more consistently distributed throughout the year” rather than prior block schedules in which larger elective blocks may have been widely or irregularly distributed. See Table 1 for representative quotes.

**Table 1:** Representative quotes

Theme	Representative quote
Improved educational experience	“As an intern, I’m still trying to figure out my role on inpatient and outpatient. It was nice to have them separate and not having to juggle since I was new to both of these roles.” “It was challenging to senior inpatient then leave for clinic. One day there was a code and I had to leave my interns and go to clinic.” “[Having] done both (pre-X+Y and post-X+Y), all of our clinics on inpatient time were kind of chaotic. I was still writing inpatient notes while on outpatient clinic – not focusing on outpatient even though that’s what I want to do.”
Wellness	“Other programs get stuck on inpatient for long periods of time. We know we will get a break. Good for mental health in general.” “I think for me overall, there are clearly a lot of benefits and some drawbacks. But the benefit to our mental health is the best. This is a huge game changer. There isn’t a lot that helps our mental health. There are pizza parties and yoga, but this really helps.” “More control/knowledge over time off. When weekends off and lighter weeks.” “You had to leave to go to clinic and sometimes go BACK to inpatient. That is unsustainable. That was your early day and then makes it your longer day.”
Longitudinal time for career development	“Knowing what half days you are off [is] good for scheduling meetings. [Can] set this afternoon aside to study or work on this project.” “A portion is continuity [clinic] and a portion is elective . . . nice since elective time is sometimes scarce as med peds.”
Scheduling difficulties created due to a mismatch between categorical X+Y schedules	“Have your categorical programs engage. . . to have standard approach.” “[should have a] schedule more congruent medicine and peds. Yeah I think that’s the biggest thing. Less room for error.”
Y block saturated with experiences	“It’s a place a lot of extra things get crammed as opposed to inpatient. A lot of structured didactic time that takes place of sleeping or going to doctors’ appointments.” “Y is not as relaxed because you have to catch up on everything.”

One perceived challenge of X+Y was related to the mismatch between categorical program schedules, leading to more challenging switches for Med-Peds residents. This specifically occurred when categorical programs use either different X+Y scheduling templates (e.g., 4+1 vs. 6+2) or different start dates of rotations (e.g., Pediatrics switch on Monday and Internal Medicine switch on Saturday). Participants felt that Med-Peds programs needed to exert particular caution to avoid overloading Y blocks with content to minimize the conflicts between competing priorities of continuity clinics, didactics, and other longitudinal experiences.

## Discussion

X+Y schedules create potential solutions for longstanding barriers to medical education, most notably conflict between inpatient and outpatient responsibilities. These barriers contribute to exhaustion among residents, and many of the early X+Y benefits demonstrate the expected improvements in these areas without resident perception of harm to the educational experience, as seen both in our results and in studies in Pediatrics residency programs [8]. While our programs have evaluated practical considerations in implementing X+Y scheduling for combined programs, it is important to capture the perception of Med-Peds residents with X+Y scheduling [10].

These larger categorical data from the AIRE pilot and our focus groups show some consistency with categorical program experience with X+Y scheduling such as improvement in educational experiences and wellness. The lack of transition within a rotation between the inpatient setting and outpatient setting (as occurs with traditional weekly half-day continuity clinic) in X+Y scheduling leads to decreased interruptions in care to attend to issues in the other setting, and the regular occurrence of ambulatory Y blocks adds predictability to the overall residency schedule. Longitudinal time for career development was also perceived favorably by Med-Peds residents. While Med-Peds residents’ experience with X+Y scheduling demonstrates perceived benefits in these areas, the downsides identified offer some unique considerations specifically for Med-Peds programs seeking to implement X+Y scheduling. First, switches may be particularly challenging with X+Y scheduling should categorical programs utilize different X+Y templates or rotation switch days, and the switch from outpatient to inpatient should be planned thoughtfully. While the Y block may be a source of flexibility for additional clinical experiences and education, during Med-Peds training in which both adult and pediatric educational experiences are being integrated, Y blocks can become excessively content-dense. Residents expressed a need to prioritize continuity clinic time during ambulatory blocks and minimize conflict with other requirements. Importantly, though the details of X+Y implementation may be different across institutions [10], these themes are relevant no matter what specific X+Y templates or clinic structure is utilized. 

Importantly, Med-Peds residents at programs where X+Y is implemented in Internal Medicine and not in Pediatrics indicated a strong preference for X+Y scheduling. These residents experienced both systems in real-time and perhaps indicate the strongest evidence for X+Y schedules. Other literature has suggested that X+Y schedules require additional resources, however, and additional factors such as backup pool availability, night schedules, and percentage of time on scheduled elective rotations may impact overall resident experience significantly [7,10].

The obvious limitation of this data is the small sample size. However, there is limited existing data for combined trainees; thus, it would be useful to use data from this qualitative program evaluation to incorporate into quantitative assessments of the themes described. Additionally, our findings are subject to recency and confirmation bias depending on when programs started an X+Y schedule, relative exposure to both scheduling systems, and knowledge of prior data related to X+Y schedules.

With more Med-Peds programs considering X+Y scheduling, whether in partnership with categorical programs or in isolation, future work should focus on verifying this initial data to inform scheduling for combined residents.

## Conclusions

X+Y schedules prove effective in mitigating conflicts between inpatient and outpatient responsibilities, addressing enduring challenges in medical education. Our data demonstrates comparable advantages of X+Y scheduling for combined residents as for their categorical counterparts. It also sheds light on some unique considerations for combined programs and trainees, including the coordination of variable categorical program X+Y models and competing priorities during ambulatory Y blocks. Ongoing innovations to meet ACGME requirements and address broad educational needs, while maximizing time in the continuity clinic, may be beneficial. These efforts continue to build on the successes of X+Y scheduling and ACGME mandates, aiming to develop scheduling models that optimize resident education, patient care, and overall wellness.

## Appendices

Interview script

Thank you for agreeing to participate in this focus group about X+Y scheduling. You were invited to participate because you are currently a resident in the program.

We are doing focus groups at the five Med-Peds programs that utilize X+Y scheduling in both medicine and pediatrics.

As a reminder, to protect your identity as a research subject, you can change your name you are logged in with and/or keep your camera off. But we will only be using the audio recordings from these sessions.

During the course of this focus group, I’ll be asking questions our team developed and may follow up with more probing questions. If you are uncomfortable with the question, you do not have to answer it. If at some point during the hour you feel you want to exit, I want to assure you that your participation is voluntary and will not impact you in any way. 

In the event that there is a breach of confidence from participants in the focus groups, residents may feel emotional distress or embarrassment by something they relay in the discussion. I want to remind everyone that the discussion occurring in the focus group needs to remain confidential and not be shared outside this event. Even though we will emphasize to all participants that comments made during the focus group session should be kept confidential, it is possible that participants may repeat comments outside of the group at some time in the future. Therefore, we encourage you to be as honest and open as you can, but remain aware of our limits in protecting confidentiality.

If you have questions or concerns about your rights as a research subject, you may contact the IRB. You can also contact me/your PD/chief directly.

Although this is a video focus group, we will only be using the audio recording from this session to obtain an accurate transcript of the discussion. A verbatim transcript will be obtained from the audio recording. I will go through it to further anonymize the data if for some reason you use a name during the hour. Once the transcript is verified, the recording will be deleted. Once that is done, the transcript will and reflections will be analyzed along with focus group data from other institutions involved in this study.

X+Y focus group questions

For Facilitators

Please read the introduction script prior to asking the questions.

1) What are the benefits you see in X+Y scheduling?

2) What are the downsides of X+Y scheduling?

3) What effect does X+Y scheduling have on your inpatient care?

4) What effect does X+Y scheduling have on outpatient care?

a. In your continuity clinic(s)?

b. On outpatient experiences that are not continuity clinic?

5) What effect does X+Y scheduling have on wellbeing? Mentorship? Career development opportunities?

6) What do you think may be the unique experience of X+Y scheduling for Med-Peds residents in comparison to your categorical colleagues?

7) What could improve your Y (ambulatory) block experiences?

8) What should the next steps in improving scheduling be? In order to improve X+Y scheduling for future classes, I would recommend _________.
